# De novo mutations in *ANK1* and *SPTB* cause hereditary spherocytosis: three case reports and literature review

**DOI:** 10.1007/s00277-026-06958-6

**Published:** 2026-04-01

**Authors:** Yumei Qin, Liuting Lu, Xiaojing Huang, Wei Li, Yanming Qin, Shifu Tang, Shaojie Wei

**Affiliations:** 1 Department of Laboratory Medicine, Key Laboratory of Precision Medicine for Viral Diseases, Guangxi Health Commission Key Laboratory of Clinical Biotechnology, Liuzhou People’s Hospital, Liuzhou, 545006 China; 2 Department of Hematology, Liuzhou People’s Hospital, Liuzhou, 545006 China; 3 Department of Laboratory Medicine, Guangxi Zhuang Autonomous Region Honorary Military Personnel Rehabilitation Hospital, No. 181, Rongjun Road, Liuzhou, Guangxi Zhuang Autonomous Region 545005 China

**Keywords:** Hereditary spherocytosis, *ANK1*, *SPTB*, de novo mutation

## Abstract

Hereditary spherocytosis (HS) is characterized primarily by jaundice, anemia, splenomegaly (enlarged spleen), increased numbers of spherocytes in peripheral blood, and elevated erythrocyte osmotic fragility. However, these manifestations are nonspecific, resulting in misdiagnosis or underdiagnosis. Here, we report three Chinese patients with a family history of HS. Case 1 was an infant who presented with jaundice and anemia during the neonatal period, without splenomegaly; case 2 was a woman with hepatitis C and mild anemia; and case 3 was a child with no obvious clinical symptoms. Genetic testing revealed a heterozygous *ANK1* c.4429 C > T (p.Arg1477*) de novo mutation in case 1, a heterozygous *ANK1* c.1025_1028dup (p.Val344Profs*13) de novo mutation in case 2, and a heterozygous *SPTB* c.5898 C > T (p.Gly1966 =) de novo variant in case 3. Interestingly, to the best of our knowledge, the *ANK1* c.4429 C > T (p.Arg1477*) and c.1025_1028dup (p.Val344Profs*13) de novo mutations have not been previously reported in public mutation databases, extending the mutation spectrum of ANK1. Overall, HS should be considered in patients with unexplained jaundice or anemia, even in those without a family history. Clinicians should conduct genetic testing to establish a definitive diagnosis, guide treatment modalities, and provide genetic counseling to patients.

## Background

Hereditary spherocytosis (HS) is a common inherited hemolytic disorder caused by mutations in genes encoding erythrocyte membrane and cytoskeletal proteins [1]. The known causative genes of HS include *ANK1*, *SPTB*, *SPTA1*, *SLC4A1*, and *EPB42*, encoding ankyrin, β-spectrin, α-spectrin, band 3 protein, and protein 4.2, respectively [1, 2]. In clinical settings, HS primarily manifests as anemia, jaundice, and splenomegaly, with an increased abundance of spherocytes in peripheral blood smears [3]. This disease has a global presence, with the prevalence varying based on ethnicity. Northern European countries have the highest incidence (about 1:2,000) [4], with cases also reported in North Africa, East Asia, India, and South America. Recently, the number of reported HS cases in China has increased, with a prevalence of about 1.38:100,000 [3, 5].

Owing to its high degree of clinical heterogeneity, the diagnosis of HS is challenging. Patients with mild HS may present with atypical or even no symptoms. In contrast, moderate to severe cases frequently present with anemia, jaundice, and splenomegaly, with long-term transfusion therapy in some cases [2]. This heterogeneity is associated with the type of membrane protein defect and the presence of comorbidities [6, 7]. Nevertheless, HS can be diagnosed across a wide age range, from the neonatal period to adulthood [8]. In patients without a family history, factors such as persistent jaundice, anemia, and splenomegaly should be carefully considered as key diagnostic indicators.

## Case presentation

### Case 1

A male infant aged 1 month and 23 days; Zhuang ethnicity; Guangxi, China. The patient was admitted to the hospital owing to pallor for more than 1 month since birth, which worsened for 2 days. The infant had pallor and jaundice since birth. At another hospital, he was diagnosed with low birth weight, prematurity, neonatal pathological jaundice, and neonatal anemia and received transfusions, anti-infective therapy, and phototherapy. He was discharged after clinical improvement. Two days before admission, progressive pallor recurred; this prompted outpatient referral to our hospital. Examination revealed the patient to be pale, with no apparent jaundice in the skin or sclera, no lymphadenopathy, normal cardiopulmonary findings, and no palpable liver or spleen below the costal margin.

### Case 2

31-year-old woman; Han ethnicity; Guangxi, China. The patient was admitted to our hospital with a 5-year history of hepatitis C and 3 days of jaundice. Five years ago, a routine pre-employment examination revealed abnormal liver function. Further evaluation resulted in the diagnosis of hepatitis C, splenomegaly, and hemolytic anemia. She received oral sofosbuvir for 4 months. Thereafter, she tested negative for HCV RNA; therefore, the treatment was discontinued as per medical advice. Three days before admission, she developed jaundice, dyspnea, and dark urine. Physical examination revealed a chronically ill appearance, marked jaundice of the skin and sclera, no lymphadenopathy, normal cardiopulmonary findings, a non-palpable liver, and a palpable spleen 5.0 cm below the costal margin.

### Case 3

8-year-old boy; Zhuang ethnicity; Guangxi, China. He was admitted for a cough for 8 days. During hospitalization, laboratory examination revealed increased total and indirect bilirubin levels, increased urobilinogen and urinary bilirubin levels, and dark urine. The patient had a history of neonatal hyperbilirubinemia, which was treated with phototherapy. Physical examination revealed a well-appearing child with a rosy complexion, no evident jaundice in the skin or sclera, no lymphadenopathy, normal cardiopulmonary findings, a non-palpable liver, and a palpable spleen 3.0 cm below the costal margin. Table 1 summarizes the laboratory findings of the three probands. All parents were healthy and non-consanguineous. They had no history of anemia or jaundice and presented with normal laboratory results.

**Table 1:** Clinical and laboratory features of 3 probands with newly diagnosed HS variants in China

Tests	Proband 1	Proband 2	Proband 3	Reference
Age	1M23D	31Y	8Y7M	NA
Gebder	Male	Female	Male	NA
Blood transfusion	Yes	No	No	NA
Splenomegaly	No	Yes	Yes	NA
Splenectomy	No	No	No	NA
RBC (×10^12^/L)	3.06	3.59	4.37	3.30–5.70
Hb (g/L)	84	91	121	97–183
Hct(%)	25.1	26.4	34.6	28.0–52.0
MCV(fL)	81.9	73.5	79.2	73.0–104.0
MCH(pg)	27.3	25.3	27.6	24.0–37.0
MCHC(g/L)	334	345	349	309–363
Ret(%)	5.96	15.93	7.69	0.5–1.5
TBIL (µmol/L)	24.1	100.7	40.6	5.1–19
IBIL (µmol/L)	13.1	90.3	27.4	1.7–12
Spherocytes (%)	0.5	18	15	NA
Hemolysis begins(g/L)	4.6	5.2	5.2	3.80–4.60
Hemolysis complete(g/L)	2.8	3.6	2.8	2.80–3.20

*Abbreviations*: *Y* year(s), *M* month(s), *D* day(s), *NA* not applicable, *RBC* red blood cell, *Hb* hemoglobin, *Hct* hematocrit, *MC*V mean corpuscular volume, *MCH* mean corpuscular hemoglobin, *MCHC* mean corpuscular hemoglobin concentration, *Ret* reticulocyte, *TBIL* total bilirubin, *IBIL*  indirect bilirubin

### Genetic analysis

The Ethics Committee of Liuzhou People’s Hospital, affiliated with Guangxi Medical University, approved this study (KY-2024-004-01). All participants provided written informed consent. Peripheral venous blood (2 mL) was collected in EDTA tubes from patients and their parents, and the samples were transported to KingMed Diagnostics Laboratory in Guangzhou for genetic testing. A QIAamp DNA Blood Mini Kit (Qiagen GmbH, Germany) was used to extract genomic DNA. A PCR-free method was used to develop the sequencing libraries. The Illumina NovaSeq 6000 platform was used to conduct whole-exome capture and high-throughput sequencing. The GATK software suite was used to analyze the sequencing data. BWA was used to align reads to the UCSC hg19 reference genome, and VEP was used to annotate variants. The variants were further filtered, pathogenicity predicted, and classified based on genetic disease databases such as ClinVar, OMIM, and HGMD; variant databases; and large-scale population sequencing databases such as gnomAD. PCR-Sanger sequencing was conducted to validate candidate variant sites. The primers used were designed using Premier 5.0 software and were as follows: *SPTB*, Forward 5’-GTGTAGCCCCGATCTCCAT-3’, Reverse 5’-GGCCAGAGTGACCCAGAGT-3’; *ANK1*, Forward 5’-TCCTGAATCTCCATGCTCG-3’, Reverse 5’-ACCTTCTCCAGCAGCACC-3’.

**Fig. 1:**
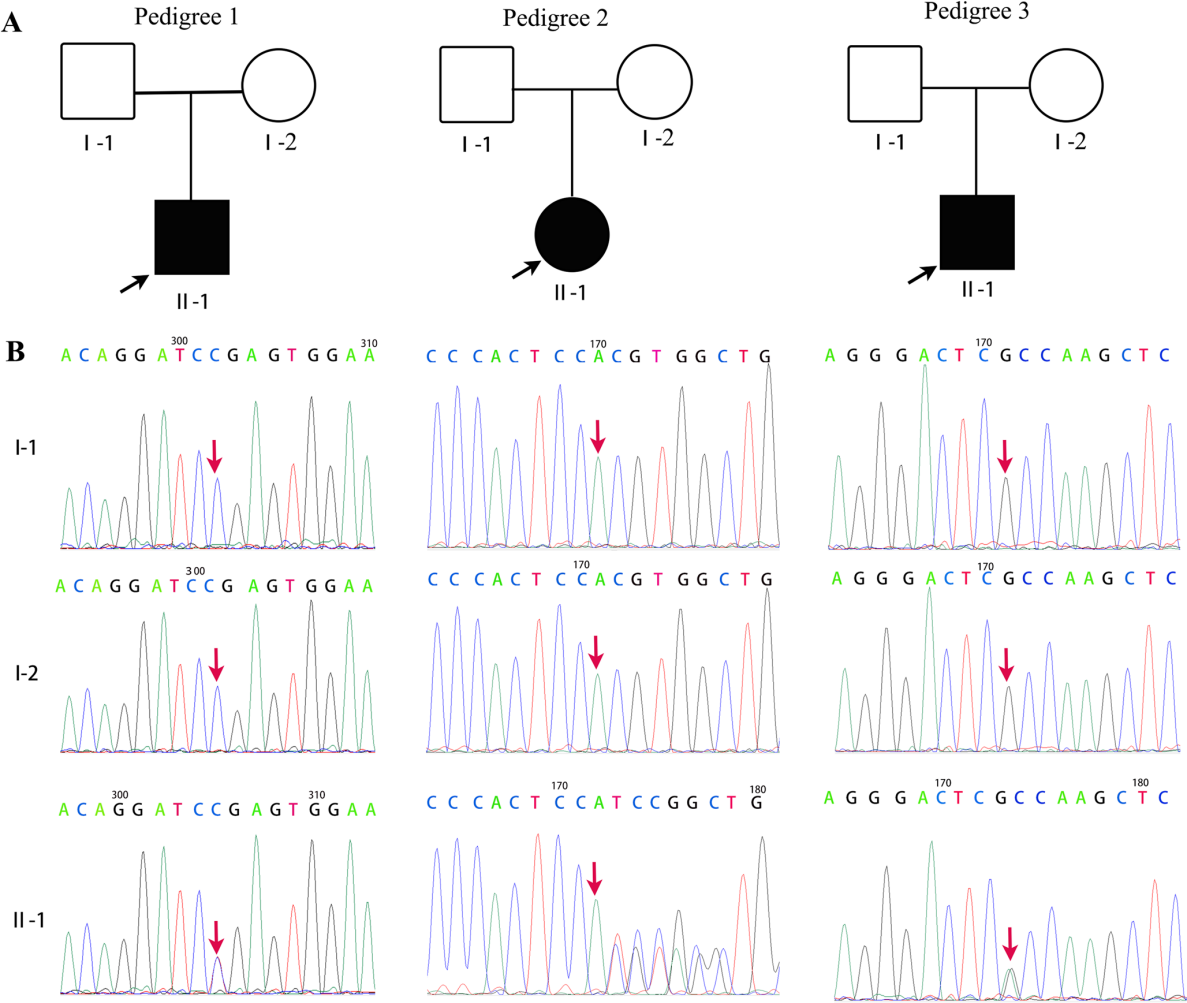
De novo mutations in ANK1 and SPTB were identified in three trios. **A**)Pedigree chart showing familial relationships and inheritance patterns. Squares denote males, circles denote females, and the shaded symbol represents the proband. **B** Sanger sequencing

**Fig. 2:**
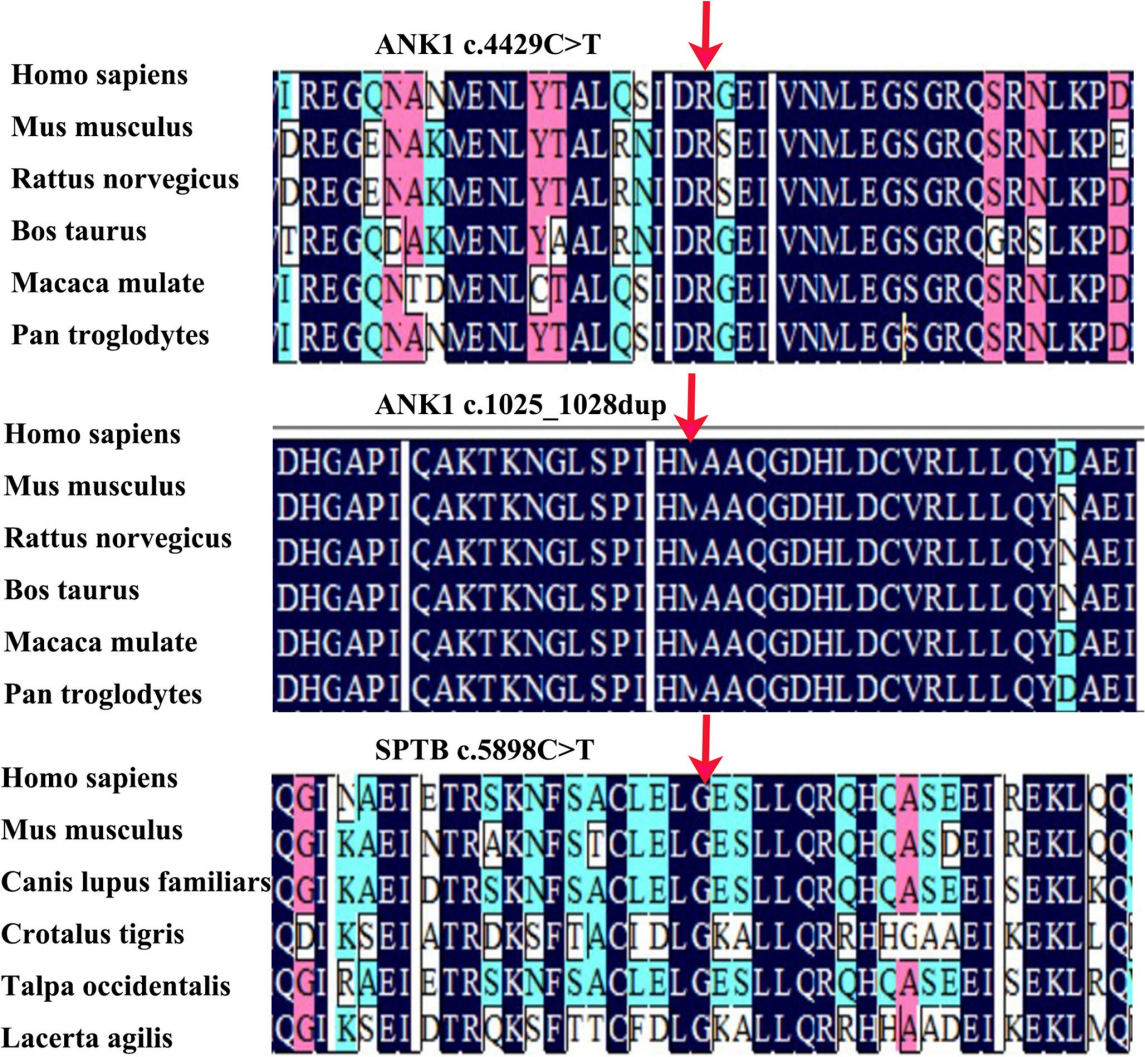
Conservation analysis of the ANK1 c.4429 C > T and c.1025_1028dup variants and the SPTB c.5898 C > T mutation sites across species. The red arrow indicates the highly conserved amino acid position affected by the mutation

Genetic testing revealed that case 1 harbored a heterozygous *ANK1* c.4429 C > T (p.Arg1477*) mutation (NM_001142446; exon 37). This mutation was classified as a de novo pathogenic variant according to the American College of Medical Genetics and Genomics guidelines (evidence: PVS1, PS2, and PM2). The parents were wild-type at this locus (Fig. 1A, B). Case 2 harbored a heterozygous *ANK1* c.1025_1028dup (p. Val344Profs*13) mutation (NM_000037.4). It was classified as de novo pathogenic (evidence: PVS1, PS2 and PM2). The parents were wild-type at this locus (Fig. 1A, B). Case 3 harbored a heterozygous *SPTB* c.5898 C > T (p.Gly1966 =) mutation (NM_001355436.2; exon 28). It was classified as a de novo variant of uncertain clinical significance. The parents tested negative for this variant (Fig. 1A, B). The mutations *ANK1* c.4429 C > T (p.Arg1477*) and c.1025_1028dup (p.Val344Profs*13) had not been previously reported in the HGMD, 1000 Genomes, or ExAC databases, extending the mutation spectrum of *ANK1*. Amino acid conservation analysis using DNAMAN software revealed high conservation of the mutated residues across different species (Fig. 2).

## Discussion

HS is a common inherited hemolytic disorder; most cases are autosomal dominant, with few being autosomal recessive [1]. The typical clinical manifestations include anemia, jaundice, and splenomegaly. Laboratory findings indicate increased numbers of spherocytes and reticulocytes and increased erythrocyte osmotic fragility; some patients also present with cholelithiasis or biliary obstruction [2, 7, 9]. However, HS exhibits significant clinical heterogeneity, resulting in misdiagnosis and underdiagnosis. Cortesi et al. [6] reported that the typical clinical manifestations of HS are frequently observed in older children and adults but are rare in neonates. Most infants with HS do not present with anemia during the first week after birth and generally do not have splenomegaly. The most common initial symptom is jaundice, and typical spherocytes are often challenging to observe in peripheral blood, making it difficult to diagnose this condition in neonates [6, 10]. In the present study, case 1 was an infant who primarily presented with jaundice and anemia. The peripheral blood reticulocyte count increased; however, spherocytes were occasional, the osmotic fragility test was negative, and no splenomegaly was observed, resulting in an atypical presentation of classic HS. Case 2 presented with hepatitis C, splenomegaly, and mild anemia. Her anemia and abnormal bilirubin metabolism were long linked to chronic liver disease and post-viral secondary changes, resulting in years of missed diagnosis of congenital hemolytic anemia. Case 3 exhibited no evident clinical symptoms and was incidentally observed to have increased total and indirect bilirubin levels, spherocyte counts in peripheral blood, and erythrocyte osmotic fragility during hospitalization for a cough. The parents of all three probands were asymptomatic and had normal laboratory results and negative family histories. Genetic testing revealed that case 1 harbored a heterozygous pathogenic *ANK1* c.4429 C > T (p.Arg1477*) mutation, which was not detected in either parent, suggesting a de novo mutation. Case 2 harbored a heterozygous *ANK1* c.1025_1028dup (p. Val344Profs*13) de novo mutation. The parents were wild-type at this locus. Case 3 harbored a heterozygous *SPTB* c.5898 C > T (p.Gly1966 =) mutation, which was not detected in either parent, suggesting a de novo variant. Therefore, HS should be considered in patients with unexplained jaundice and anemia. Furthermore, family investigations combined with genetic testing should be performed to establish an early and definitive diagnosis.

To date, the HGMD (www.hgmd.cf.ac.uk, last accessed: April 2024) has listed 389, 384, 214, 262, and 32 mutations in *ANK1*, *SPTB*, *SPTA1*, *SLC4A1*, and *EPB42*, respectively. *ANK1* and *SPTB* are the most frequently mutated genes in HS; however, they exhibit diverse and complex mutation spectra with no recognized hotspots. This background underscores the importance of reporting these novel *ANK1* variants (c.4429 C > T and c.1025_1028dup) to expand the mutational spectrum of the disease.

With respect to the mutational spectrum and frequency of de novo mutations in HS, recent large-cohort studies—particularly in the Chinese population—have elucidated the genetic landscape of the disease. Consistent with our findings, ANK1 and SPTB are confirmed as the most frequently mutated genes, collectively accounting for approximately 70% of HS cases [11, 12]. In terms of mutation types, loss-of-function variants—including nonsense and frameshift mutations—predominate in ANK1 and SPTB, whereas missense mutations are more prevalent in SLC4A1 [12]. Notably, the two novel ANK1 mutations identified in our study (p.Arg1477* and p.Val344Profs*13) fit this established loss-of-function pattern. Regarding the frequency of de novo mutations, pedigree analyses have shown that de novo events account for a substantial proportion of HS cases. A recent study of 34 Chinese families found that 68.0% (17/25) of cases in which family segregation analysis was performed had de novo mutations [13]. Furthermore, compared with other HS-associated genes, de novo variations have been shown to occur more frequently in the ANK1 gene [12]. In our study, all three probands carried de novo mutations—two in ANK1 and one in SPTB—which aligns with these observations and highlights the significant role of de novo variants in HS pathogenesis, particularly in patients with negative family histories. It should be noted that EMA binding testing was not performed in these cases, as the diagnosis was established through genetic analysis.

The ANK1 gene is located on chromosome 8p11.21, spans about 160 kb, and contains 42 exons encoding ankyrin, a 1,880-amino-acid protein. A 215-bp fragment at the 5’ end is unique to erythrocyte ankyrin and exhibits promoter activity [14]. Mutations in *ANK1* disrupt the vertical linkage system of the ankyrin-mediated erythrocyte membrane, hindering the link between integral membrane proteins in the lipid bilayer and the underlying cytoskeletal network. This results in a marked decrease in membrane stability, loss of vesicular membrane material, decreased red blood cell surface area, spherocyte formation, decreased deformability, and ultimately hemolytic anemia [3]. The SPTB gene is located on chromosome 14q23.3, spans more than 100 kb, and contains 35 exons encoding β-spectrin, a 2,137-amino-acid protein [7, 8, 15]. β-Spectrin is a major component of the cytoskeletal network and is vital for regulating membrane deformability and mechanical stability [15]. In the present study, case 1 harbored a heterozygous *ANK1* c.4429 C > T (p.Arg1477*) nonsense mutation, a de novo pathogenic variant. This mutation converts arginine at position 1,477 to a stop codon, leading to premature termination of protein synthesis and a truncated, nonfunctional protein. Case 2 harbored a heterozygous *ANK1* c.1025_1028dup (p. Val344Profs*13) frameshift mutation, changing valine at position 344 to proline. This frameshift, owing to a non-triplet base duplication in the coding region of *ANK1*, may cause the loss of normal protein function via nonsense-mediated mRNA decay or premature termination of the coding sequence. Case 3 harbored a heterozygous *SPTB* c.5898 C > T (p.Gly1966 =) synonymous mutation, a de novo variant. The mutation changes a cytosine to a thymine at nucleotide 5,898, without altering the amino acid. This variant may disrupt or create splicing regulatory elements, potentially resulting in aberrant mRNA splicing [16]; however, functional experiments are warranted to verify its exact pathogenicity. Database searches in HGMD, 1000 Genomes, and ExAC suggested that *ANK1* c.4429 C > T (p.Arg1477*) and c.1025_1028dup (p.Val344Profs*13) have not been previously reported, expanding the mutation spectrum of *ANK1*. Analysis of amino acid sequence conservation using DNAMAN software revealed that the mutated residues in all three probands were highly conserved across different species.

## Conclusion

In summary, we report the cases of three patients with negative family histories of HS. All the patients harbored de novo mutations. To the best of our knowledge, we identified two novel pathogenic *ANK1* variants that have not been previously reported in public mutation databases. These findings underscore the importance of genetic testing in patients with unexplained jaundice and anemia, even in the absence of a family history, after common causes are excluded. Early genetic diagnosis is essential for guiding clinical treatment, developing treatment strategies, assessing prognosis, and providing genetic counseling

## Data Availability

The raw data supporting the conclusions of this article will not be made publicly available due to patient privacy and confidentiality protections under the ethical approval. However, they are available from the corresponding author upon reasonable request and with appropriate institutional approval.
